# Antibiofilm effects of punicalagin against *Staphylococcus aureus in vitro*

**DOI:** 10.3389/fmicb.2023.1175912

**Published:** 2023-04-14

**Authors:** Yunfeng Xu, Weiping Guo, Denglin Luo, Peiyan Li, Jinle Xiang, Junliang Chen, Xiaodong Xia, Qinggang Xie

**Affiliations:** ^1^College of Food and Bioengineering, Henan University of Science and Technology, Luoyang, Henan, China; ^2^School of Food Science and Technology, Dalian Polytechnic University, Dalian, Liaoning, China; ^3^Heilongjiang Feihe Dairy Co. Ltd., Beijing, China

**Keywords:** punicalagin, *Staphylococcus aureus*, antibiofilm, microscopy, hydrophobicity

## Abstract

*Staphylococcus aureus* is a common foodborne pathogen which can form biofilms to help them resist to antimicrobials. It brings great harm to human health. Punicalagin has good antimicrobial activities against *S. aureus*, but its effect on biofilm formation has not been clearly illustrated. The aim of this study was to explore the antibiofilm effects of punicalagin against *S. aureus*. Results showed that punicalagin did not significantly interfere with the growth of *S. aureus* at the concentrations of 1/64 MIC to 1/16 MIC. The biomass and metabolic activity of biofilms were significantly reduced when exposed to sub-inhibitory concentrations of punicalagin. The number of viable cells in the biofilms was also decreased after punicalagin treatment. Scanning electron microscopy and confocal laser scanning microscopy images confirmed that punicalagin damaged the structure of biofilms. The antibiofilm mechanism was partly due to the modification of the cell surface which led to the reduction of cell surface hydrophobicity. These findings suggest that punicalagin has the potential to be developed as an alternative to control *S. aureus* biofilms.

## Introduction

Most bacteria adhere to a surface in the form of biofilm in nature. In comparison with planktonic bacteria, biofilm bacteria have stronger resistance to disinfectants, ultraviolet rays, heavy metals, antibiotics, acids, alkalis and salts owing to genetic and metabolic adaptations (Phuong et al., 2017). Biofilm also brings great potential safety hazards to food industry. Some pathogenic bacteria can colonize, grow and form biofilm on food surface, food processing machinery surface and pipeline, food processing environment and packaging materials, which often lead to pipeline corrosion or product pollution, thus bringing huge economic losses. *Staphylococcus aureus* is a common pathogen which can form biofilm and bring serious safety risks to medical and food fields. There is an urgent need to search for safe and effective antimicrobials for the treatment of the bacteria.

In recent years, many researchers have turned their attention to natural products, especially plant-derived active substances (Bouarab Chibane et al., 2019). Punicalagin, the main component of pomegranate peel polyphenols, is a high molecular weight (1084.72) water-soluble compound (Aqil et al., 2012). It is known for various biological properties, including antioxidant, anti-inflammatory, anti-cancer and immunomodulatory activities (Benchagra et al., 2021; Berdowska et al., 2021; Čolić et al., 2022; Xu et al., 2022). It has also been reported to possess antimicrobial activities against several pathogenic bacteria such as *S. aureus*, *Salmonella*, and *Vibrio parahaemolyticus* (Li et al., 2014; Mun et al., 2016; Liu et al., 2022). Our previous study has shown that punicalagin exhibited a good bacteriostatic effect on *S. aureus* with the minimum inhibitory concentration (MIC) of 0.25 mg/ml (Xu et al., 2017). However, the effect of punicalagin on the biofilm formation of *S. aureus* and its action mechanism is unclear. Therefore, this study was aimed to investigate the antibiofilm effects of punicalagin against *S. aureus*. It provides a theoretical and experimental basis for the development and utilization of punicalagin and the control of *S. aureus* biofilm.

## Materials and methods

### Bacterial strains and cultural conditions

*Staphylococcus aureus* ATCC 29213, obtained from the American Type Culture Collection, was stored in tryptone soybean broth (TSB; Land Bridge Technology Co. Ltd., Beijing, China) containing 25% glycerol at −80°C. It was taken out from the refrigerator and inoculated on tryptone soybean agar (TSA; Land Bridge Technology Co. Ltd., Beijing, China) plate before each experiment. Single colony of the strain was picked out, inoculated into TSB, and cultured at 37°C for about 12 h. The absorbance of the bacterial suspension was measured by a spectrophotometer (Smart Spec™ plus, Bio-Rad Laboratories, Hercules, CA) to obtain the optical density about 0.5 at 600 nm.

### Growth curves

The growth curves of *S. aureus* were detected when treated with or without punicalagin using the Bioscreen C automated microbiology growth curve analysis system (Labsystems, Helsinki, Finland) as described by Zheng et al. (2020). Punicalagin (≥98%, CAS 65995–63-3, Must Bio-Technology Co. Ltd., Chengdu, China) was added into the wells to obtain the final concentrations of 0 (control), 1/64, 1/32, 1/16 and 1/8 MIC, respectively. The above bacterial suspension was inoculated at a ratio of 1% containing about 10^6^ colony-forming units (CFU)/mL. Sterile TSB containing corresponding concentrations of punicalagin was taken as negative control. The microplate was incubated statically at 37°C and the absorbance at 600 nm wavelength was measured in 1 h intervals for 24 h.

### Biofilm formation assay

The effect of punicalagin on biofilm biomass was conducted by crystal violet staining method referred to Fan et al. (2022b). Briefly, *S. aureus* suspensions exposure to punicalagin at 0 (control), 1/64, 1/32 and 1/16 MIC were incubated in a 96-well microplate at 37°C for 24 h. After removing the planktonic cells, wells were washed with sterile phosphate buffered saline (PBS) for three times and fixed with methanol for 15 min. Then crystal violet solution (1%) was added to stain biofilms and the wells were rinsed thrice with distilled water, followed by the addition of acetic acid (33%, vol/vol). After shaking at low speed for 5 min, the absorbance at 570 nm was measured using a microplate reader (model 680; Bio-Rad). The relative biofilm formation was calculated by the OD_treatment_ normalized with OD_control_.

### Biofilm metabolic activity assay

The effect of punicalagin on biofilm metabolism was examined by the method of Jadhav et al. (2013). The prepared bacterial suspensions containing different concentrations of punicalagin (0, 1/64, 1/32 and 1/16 MIC) were separately added to a 96-well plate and incubated at 37°C for 24 h. Then the bacterial suspensions were removed. The plate was gently rinsed three times with PBS. A total of 250 μl of 3-[4, 5-dimethylthiazol-2-yl]-2, 5-diphenyltetrazolium bromide (MTT; Beijing Solarbio Science and Technology Co., Ltd., Beijing, China) solution at the concentration of 0.5 mg/ml was added to each well, and incubated at 37°C for 3 h. The insoluble purple formazan was further dissolved in dimethyl sulfoxide (DMSO) and the absorbance was measured at a wavelength of 570 nm using a microplate spectrophotometer (model 680; Bio-Rad).

### Counting of viable bacteria in biofilms

The number of viable bacteria in biofilms was counted as previously reported with some modifications (Amalaradjou and Venkitanarayanan, 2011). Briefly, the bacterial suspensions containing different concentrations of punicalagin (0, 1/64, 1/32 and 1/16 MIC) were inoculated into a 24-well polystyrene plate at 2 ml per well and cultured at 37°C for 24 h. Then the wells were carefully washed three times with sterile PBS, followed by the addition of another 2 ml of PBS. The biofilms were wiped off and mixed thoroughly. After a series of 10-fold dilution, the suspensions were spread on TSA, and cultured at 37°C overnight before the colonies were counted.

### Field-emission scanning electron microscopy observation

FESEM was performed as described by Li et al. (2021) with some modifications. The bacterial suspensions containing punicalagin at different concentrations (0, 1/64, 1/32 and 1/16 MIC) were added to a 12-well plate pre-placed with sterile glass sheets. After incubated at 37°C for 24 h, the glass sheets were rinsed gently with 2 ml of PBS for three times to wash off the planktonic bacteria. Then they were placed in 2.5% glutaraldehyde solution (prepared in PBS) at 4°C for 5 h, followed by fixation with 1% osmic acid solution for 5 h. Samples were washed with PBS and dehydrated with different concentrations of ethanol solution (30, 50, 70, 80, 90 and 100%). After naturally air dried in a fume hood overnight, the samples were immobilized on a support, and sprayed with Au-Pd under vacuum. Finally, the morphology of the biofilm was observed and photographed by a scanning electron microscope (S-4800, Hitachi, Tokyo, Japan).

### Confocal laser scanning microscopy observation

CLSM was carried out according to Fan et al. (2022a) with minor changes. As described above, *S. aureus* biofilm was formed on glass sheets with or without the treatment of punicalagin (0, 1/64, 1/32 and 1/16 MIC) at 37°C for 24 h. After gently rinsed three times with PBS, the glass sheets were transferred to a new 12-well plate. A total of 1 ml of SYTO 9 and propidium iodide (PI) fluorescent dye mixture was added to the wells, followed by the incubation at room temperature in the dark for 15 min. The biofilms were observed under a confocal laser scanning fluorescence microscope (A1; Nikon, Tokyo, Japan). Live bacteria with intact cell membranes emitted green fluorescence, while dead or damaged bacteria emitted red fluorescence.

### Determination of bacterial surface hydrophobicity

The effect of punicalagin on the cell surface hydrophobicity of *S. aureus* was assessed by the method of microbial adhesion to hydrocarbons (MATH) as reported previously (Tang et al., 2020). In brief, overnight *S. aureus* culture was collected, washed, and resuspended in PBS. Punicalagin was added to reach the concentrations of 0, 1/64, 1/32 and 1/16 MIC. The absorbance of each sample was detected at 600 nm. Then, 1 ml of hexadecane was added to 2 ml of the bacterial suspension and mixed thoroughly for 1 min. The absorbance of aqueous phase was detected again after incubated for 15 min at 37°C. The hydrophobicity was calculated as follow.


Hydrophobic rate(%)=(Aa−Ab)/Aa×100%

Where Aa is the initial absorbance at 600 nm, and Ab is the absorption value after the treatment of punicalagin. Control groups were those without punicalagin treatment.

### Statistical analysis

Mean values and standard deviations were obtained from three replicate experiments. Statistical analysis was performed by the analysis of variance (ANOVA) with SPSS 20.0 software. Tukey’s multiple range test was used to calculate the significant differences (*p* < 0.05) between the control and treatment groups.

## Results

### Effect of punicalagin on bacterial growth

**Figure 1:**
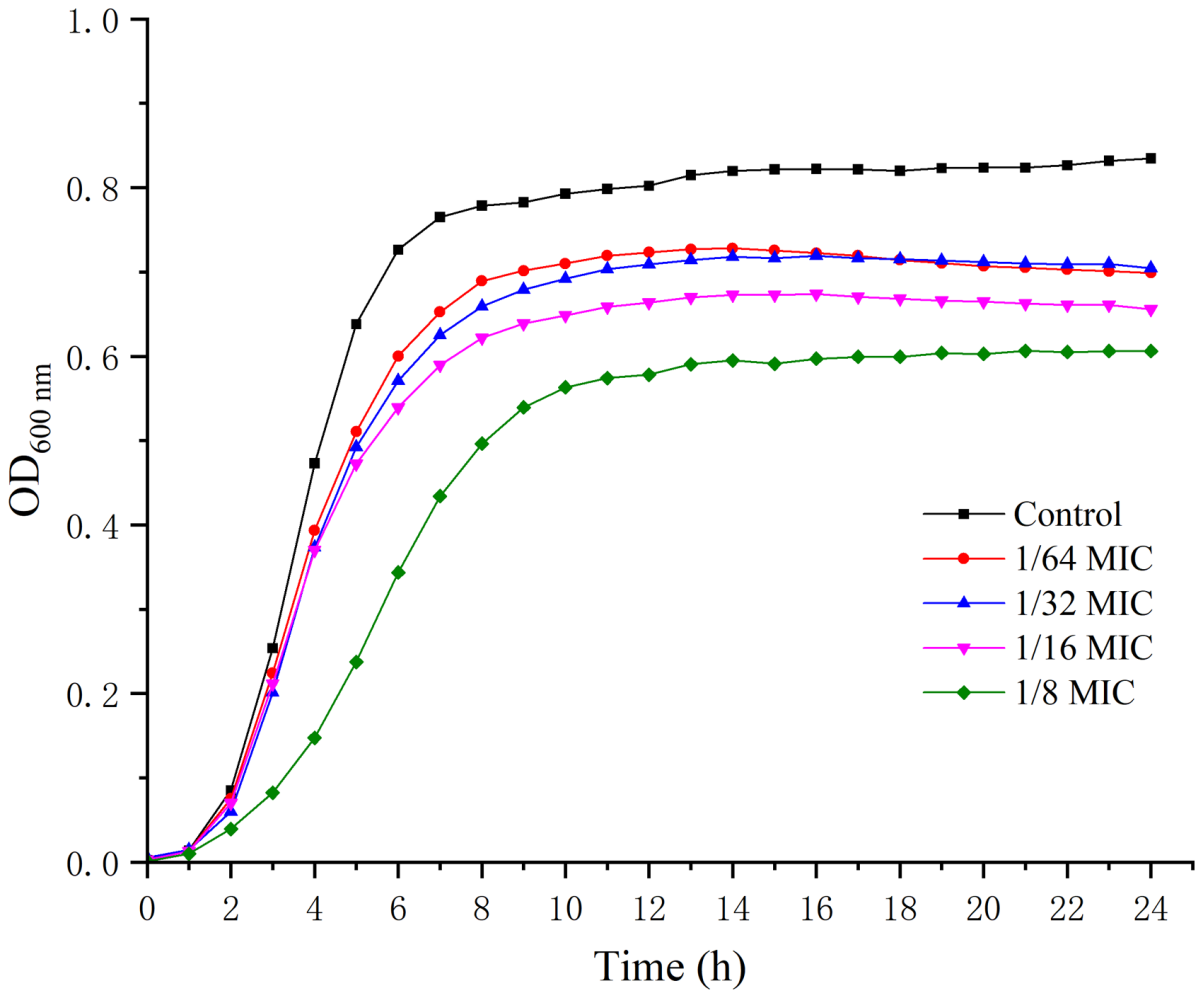
Growth curves of *S. aureus* in the absence or presence of punicalagin (0, 1/64 MIC, 1/32 MIC, 1/16 MIC and 1/8 MIC).

The effect of punicalagin on the growth of *S. aureus* was shown in Figure 1. After about 2 h of lag, the strains of control groups quickly entered the logarithmic growth phase and reached a stationary phase within 8 h. The growth of *S. aureus* was obviously inhibited by punicalagin at 1/8 MIC compared to the control. However, punicalagin has minor effect on the growth of *S. aureus* at 1/64 MIC to 1/16 MIC. Therefore, the concentrations of punicalagin from 1/64 MIC to 1/16 MIC were considered as sub-inhibitory concentrations (SICs) against *S. aureus* which were chosen for the following experiments.

### Effect of punicalagin on biofilm formation

**Figure 2:**
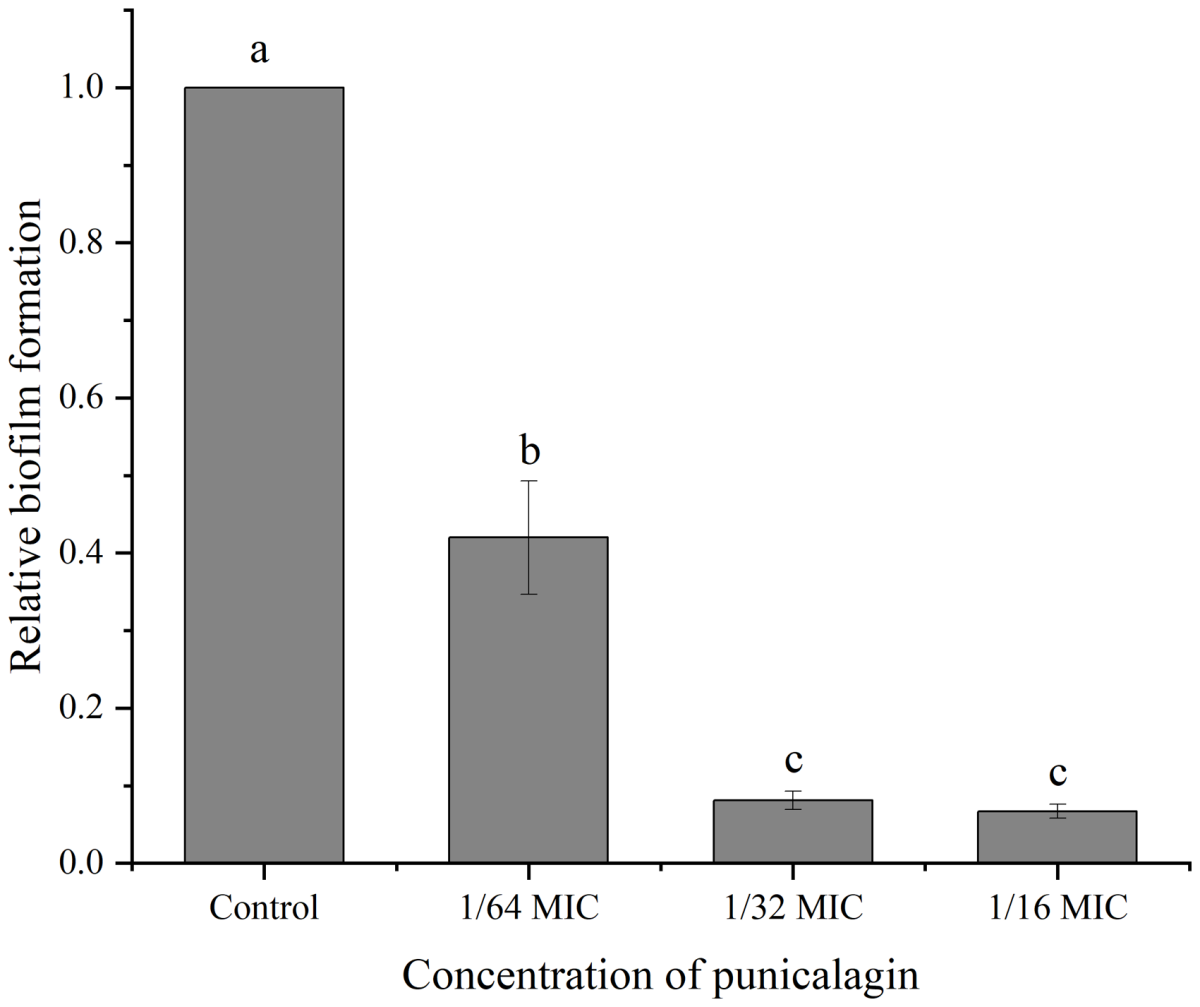
Inhibition of punicalagin (0, 1/64 MIC, 1/32 MIC, and 1/16 MIC) on *S. aureus* biofilm formation by crystal violet staining assay. Each bar indicates means ± the standard deviation. Different letters represent a significant difference compared to the control (*p* < 0.05).

As shown in Figure 2, the relative biofilm formation by *S. aureus* on polystyrene microplate was markedly decreased after punicalagin treatment. The biomass of the biofilm was decreased to 42.0% at the presence of punicalagin at 1/64 MIC compared to the control. And a higher reduction to 8.1% occurred as the concentration of punicalagin increased to 1/32 MIC. There was no significant difference between the 1/32 MIC and 1/16 MIC groups. This result indicated that punicalagin prevented biofilm formation effectively at SICs.

### Effect of punicalagin on metabolic activity of biofilms

**Figure 3:**
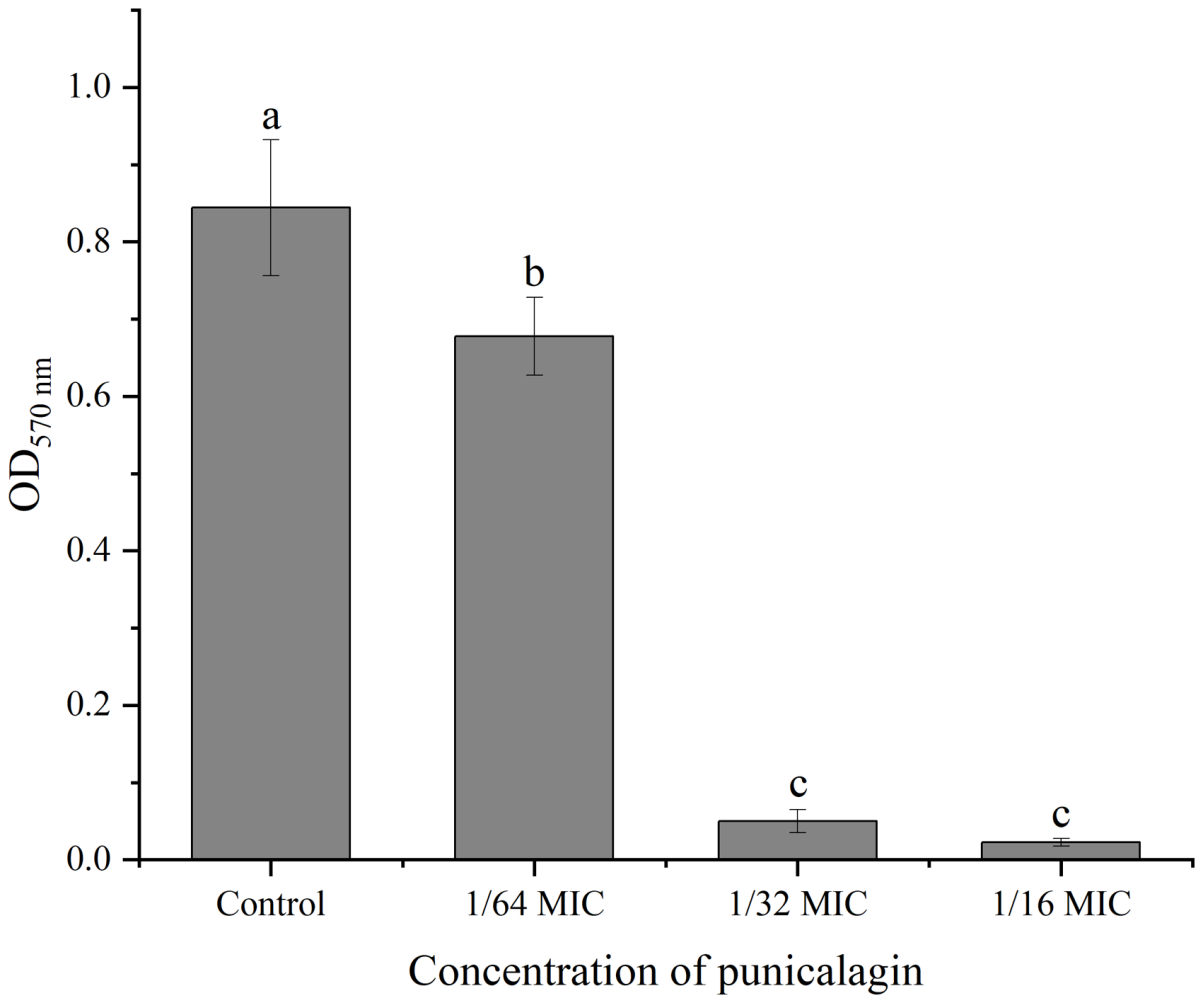
The effect of punicalagin on biofilm metabolic activity by MTT staining assay. Each bar indicates means ± the standard deviation. Different letters represent a significant difference compared to the control (*p* < 0.05).

MTT staining method reflects the metabolism of live bacteria in the biofilm and the result was depicted in Figure 3. At 1/64 MIC, punicalagin has a significant inhibitory effect on the metabolism of bacteria in the biofilm. The inhibitory effect increased with the increase of the concentration of punicalagin. At the highest concentration tested (1/16 MIC), the optical density was reduced by 97.3% in comparison with the control.

### Number of viable bacteria in biofilm

**Figure 4:**
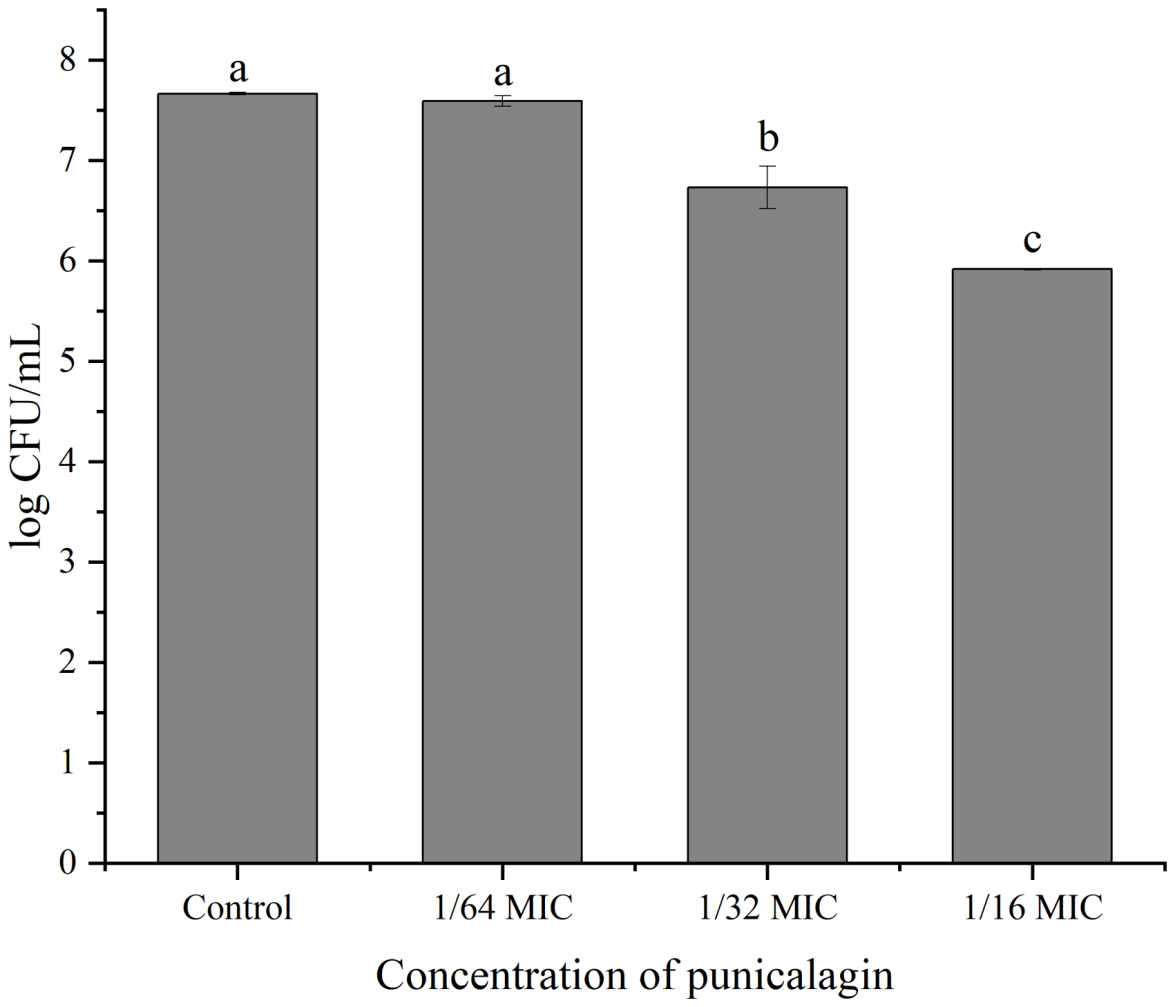
Cell enumeration of biofilms after exposed to various concentrations of punicalagin. Error bars represent standard deviations from triplicate analyzes of each sample. Different letters represent a significant difference compared to the control (*p* < 0.05).

Figure 4 shows the number of viable bacteria in biofilm. It can be observed that the biofilm-associated population of *S. aureus* was reduced by the treatment of punicalagin and higher concentrations of punicalagin lead to more reduction in viable bacteria. Specifically, the viable cell counts were decreased by 0.94 and 1.75 log CFU/mL in comparison with the control after incubated with punicalagin at the 1/32 MIC and 1/16 MIC, respectively.

### Effect of punicalagin on biofilm of *Staphylococcus aureus* under FESEM

**Figure 5:**
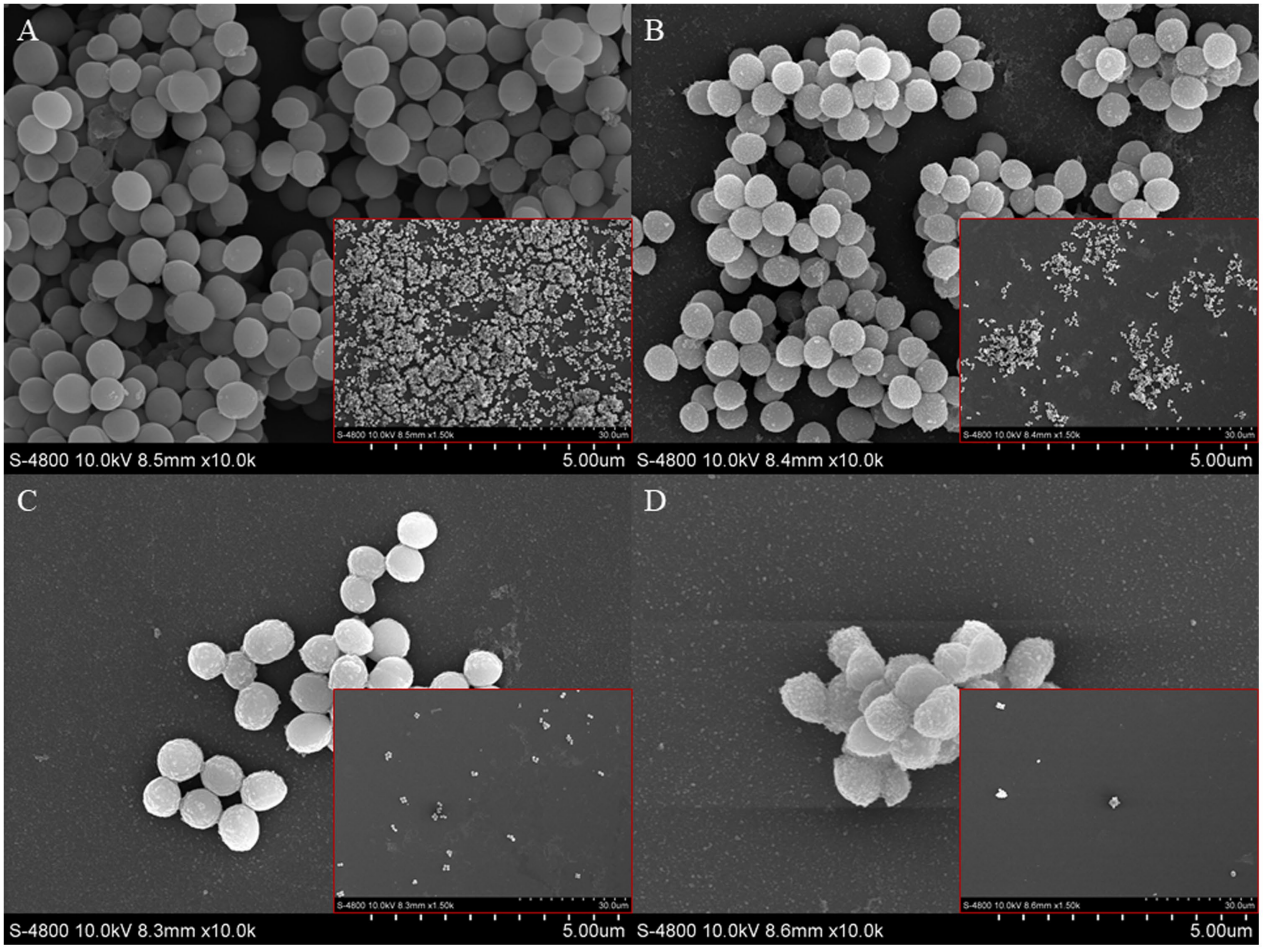
Scanning electron microscopic images of *S. aureus* biofilm in the presence of punicalagin at concentrations of 0 **(A)**, 1/64 MIC **(B)**, 1/32 MIC **(C)** and 1/16 MIC **(D)**. Biofilm images in small red boxes are at 10,000 × magnification, and the large images are 1,500 × magnified.

FESEM was used to observe the effect of punicalagin on the structure of biofilm. The results are shown in Figure 5. In Figure 5A, the cells are tightly adhered to each other on the surface of the glass slide and stacked on top of each other, suggesting the strong biofilm formation ability of the strain. The cells in Figures 5B–D are under the action of SICs of punicalagin. The adhesion between the cells became looser and the distribution became sparser than the control. This experiment visually illustrates the inhibitory effect of punicalagin on the biofilm formation of *S. aureus*. It also can be clearly seen that the surface of the control cells was smooth, while the surface of the punicalagin-treated cells was rough, indicating that punicalagin disrupted the cell surface morphology.

### Effect of punicalagin on biofilm of *Staphylococcus aureus* by CLSM

**Figure 6:**
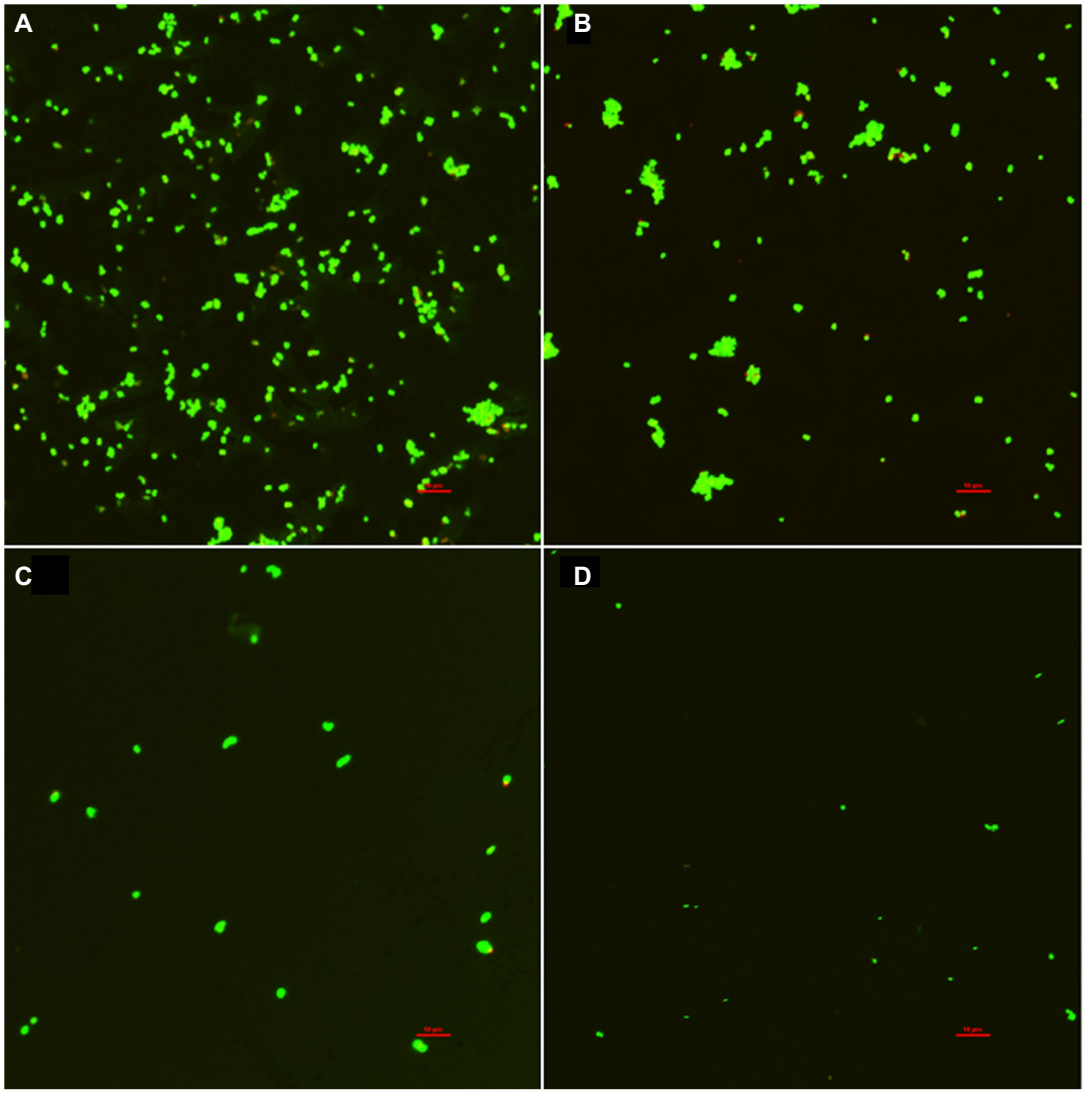
Confocal laser scanning microscopic images of *S. aureus* biofilm in the presence of punicalagin at concentrations of 0 **(A)**, 1/64 MIC **(B)**, 1/32 MIC **(C)** and 1/16 MIC **(D)**. Scale bar =10 μm.

The inhibitory effect of punicalagin on the biofilm formation of *S. aureus* was also observed by CLSM. As can be seen from Figure 6, the fluorescence intensity of the control group was significantly higher than that of the experimental group after the action of punicalagin. A large number of *S. aureus* aggregates can be seen in the control group. After the treatment of punicalagin, the ability of *S. aureus* to form biofilm was significantly reduced. The amount of biofilm formation decreased as the concentration of punicalagin increased. The proportion of viable bacteria in all groups accounted for the majority (green). Only a few bacteria emitted red fluorescence, suggesting impaired cell membranes.

### Effect of punicalagin on *Staphylococcus aureus* surface hydrophobicity

**Figure 7:**
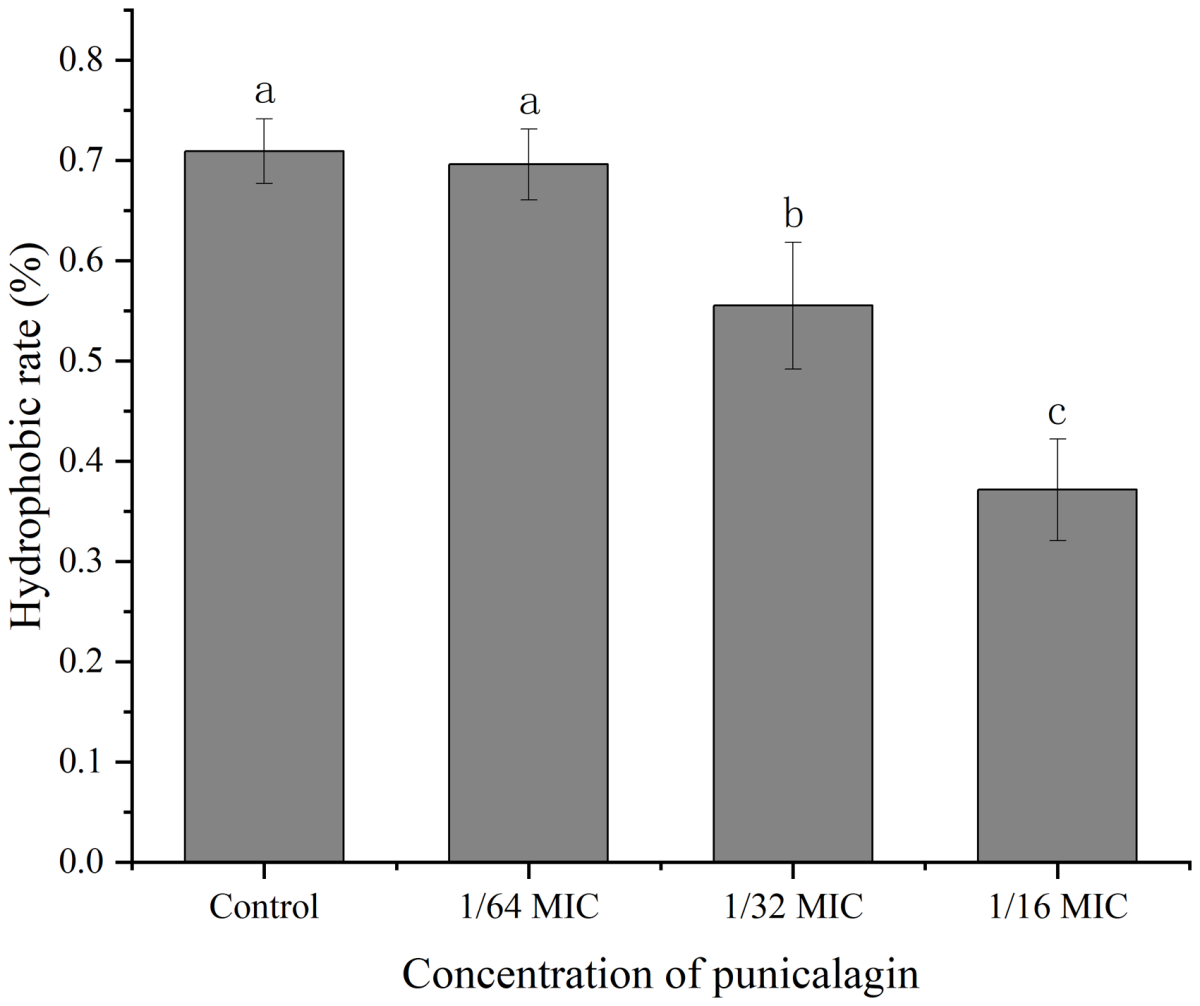
The effect of punicalagin on cell surface hydrophobicity of *S. aureus*. Error bars represent standard deviations from triplicate analyzes of each sample. Different letters represent a significant difference in comparison with the control (*p* < 0.05).

The result of bacterial surface hydrophobicity was shown in Figure 7. No significant difference was seen on the hydrophobic rates between the control and 1/64 MIC groups with the result of 70.9 and 69.6%, respectively. But the hydrophobic rates were significantly reduced to 55.5 and 37.2% after treated with 1/32 MIC and 1/16 MIC of punicalagin.

## Discussion

In this study, the antibiofilm activity of punicalagin against *S. aureus* was investigated. Punicalagin at SICs could significantly inhibit the production of biofilm biomass. Many antibiofilm agents have been reported to possess similar activities against *S. aureus*. Shikimic acid was confirmed to reduce the biomass of the biofilm dose-dependently at its sub-MICs (Bai et al., 2019). Thymol exhibited a concentration-dependent antibiofilm activity with maximum biofilm inhibition of 88% at 100 μg/ml without affecting growth (Valliammai et al., 2020). The metabolic activity of *S. aureus* biofilm was significantly reduced when treated with punicalagin from 1/64 MIC to 1/16 MIC. There are some other compounds that inhibited the biofilm formation of *S. aureus* coupled with the reduction in cellular metabolic activity of biofilm. Kannappan et al. (2017) reported the inhibitory effect on the biofilm formation and metabolic activity of *S. aureus* by *Vetiveria zizanioides* root extract. Parai et al. (2020) found reserpine stopped the metabolic activity of 50.6% bacterial cells at 1/2 MIC. Punicalagin also induced a reduction of the number of bacteria in the biofilm. But this result was not exactly the same as the biofilm biomass and metabolic activity due to the difference of experimental principles and methods.

Microscopic visualization of punicalagin induced alterations on biofilm architecture of *S. aureus* was made through FESEM analyzes. The biofilm without punicalagin treatment was observed to adhere on the surface of glass slide and form thick aggregates, while the biofilms exposed to SICs of punicalagin gradually decreased. This result was in accordance with previous reports that many natural compounds were able to destroy the structure of biofilm. For instance, Gu et al. (2022) observed the biofilm of *S. aureus* USA300 on slides decreased as the concentration of geraniol increased. The morphological changes of *S. aureus* biofilm treated with punicalagin was also confirmed by CLSM. The fluorescent images were well correlated with the measured antibiofilm effects. It was reported that kaempferol dose-dependently inhibited the biofilm formation of *S. aureus* as observed by the fluorescence microscopy (Ming et al., 2017). Similarly, a reduction in the thickness of biofilm formation was noticed in myrtenol treated samples compared to the control (Selvaraj et al., 2019).

Adhesion is the initial and key step of biofilm formation. Bacterial adhesive ability is closely relevant to cell surface hydrophobicity (Zhu et al., 2022). Generally, the higher hydrophobicity, the stronger adhesive ability. The ability of bacterial hydrophobicity was decreased by the treatment of punicalagin, which resulted in the decrease of cell attachment, and eventually interfered with biofilm formation. Wen et al. (2021) reported the significant dose-related reduction in cell surface hydrophobicity of *S. aureus* with increasing concentrations of naringenin. Wang et al. (2017) found that hydrophobic rates of *S. aureus* decreased to 32.1 and 28.1% after exposed to MIC and MBC level of *Dodartia orientalis* L. essential oil, respectively. However, the mechanism of *S. aureus* biofilm formation is complex (Peng et al., 2023). The effect of punicalagin on the expression of genes critical for biofilm formation needs to be determined in the future.

Besides, how to apply punicalagin to food industry is another unaddressed issue. Tayel et al. (2012) added the ethanol extract of pomegranate peel into the meat steaks for decontamination. Andrade et al. (2023) incorporated pomegranate peel extract (85.84 mg/g punicalagin, 6.67 mg/g ellagic acid) into polylactic acid-based packaging film to extend the shelf life of beef meat. More studies about its activity in the food system are necessitated before application.

## Conclusion

In conclusion, our study demonstrated that punicalagin exhibited good antibiofilm capacity against *S. aureus in vitro*. It reduced the biomass, metabolic activity and the number of microcolonies in the biofilm and impaired biofilm structure at SICs in a dose-dependent manner. Antibiofilm effect of punicalagin could be partly explained by the change of bacteria surface hydrophobicity. Based on these findings, punicalagin may have the potential to be developed as an antibiofilm agent against *S. aureus*.

## Data availability statement

The original contributions presented in the study are included in the article/supplementary material, further inquiries can be directed to the corresponding authors.

## Author contributions

XX conceived and designed the experiments. YX and WG performed the experiments. JC and QX analyzed the data. DL, PL, and JX contributed to reagents, materials, and analysis tools. YX wrote the manuscript. All authors contributed to the article and approved the submitted version.

## Funding

This work was supported by the Doctor Scientific Research Start-up Fund of Henan University of Science and Technology (13480067).

## Conflict of interest

QX was employed by Heilongjiang Feihe Dairy Co. Ltd.

The remaining authors declare that the research was conducted in the absence of any commercial or financial relationships that could be construed as a potential conflict of interest.

## Publisher’s note

All claims expressed in this article are solely those of the authors and do not necessarily represent those of their affiliated organizations, or those of the publisher, the editors and the reviewers. Any product that may be evaluated in this article, or claim that may be made by its manufacturer, is not guaranteed or endorsed by the publisher.
